# Detection of miR-133a-5p Using a Molecular Beacon Probe for Investigating Postmortem Intervals

**DOI:** 10.3390/ncrna10060058

**Published:** 2024-11-26

**Authors:** Eun Hye Lee, Mingyoung Jeong, Kwangmin Park, Dong Geon Lee, Eun Ju Lee, Haneul Lee, Ah Yeoung Kim, Jae Won Ahn, Hyun Jun Woo, Sunghyun Kim, Jaewon Lim, Jungho Kim

**Affiliations:** 1 Department of Forensic Science, Graduate School, Catholic University of Pusan, Busan 46252, Republic of Korea; dmsgp5484@naver.com (E.H.L.); mingom1892@gmail.com (M.J.); g_d2001@naver.com (H.L.); j4030hot@nate.com (J.W.A.); shkim0423@cup.ac.kr (S.K.); 2 Next-Generation Industrial Field-Based Specialist Program for Molecular Diagnostics, Brain Busan 21 Plus Project, Graduate School, Catholic University of Pusan, Busan 46252, Republic of Korea; pkmchi777@naver.com (K.P.); ehdrjs6789@naver.com (D.G.L.); ah_zer01@naver.com (A.Y.K.); 3 Department of Clinical Laboratory Science, College of Health Sciences, Catholic University of Pusan, Busan 46252, Republic of Korea; leeeunju41@naver.com; 4 Korea Mycobacterium Resource Center (KMRC), Department of Research and Development, The Korean Institute of Tuberculosis, Osong 28158, Republic of Korea; 5 Department of Clinical Laboratory Science, Semyung University, Jecheon 27136, Republic of Korea; taesube@semyung.ac.kr

**Keywords:** postmortem interval, microRNA, molecular beacon probe, biomarker

## Abstract

**Background:** When a body is discovered at a crime or murder scene, it is crucial to examine the body and estimate its postmortem interval (PMI). Accurate estimation of PMI is vital for identifying suspects and providing clues to resolve the case. MicroRNAs (miRNAs or miRs) are small non-coding RNAs that remain relatively stable in the cell nucleus even after death-related changes occur. **Objective**: This study developed a molecular beacon probe for mmu-miR-133a-5p and assessed its use in mouse muscle tissue at temperatures of 4 °C and 21 °C to estimate the PMI. **Methods:** A total of 36 healthy adult male BALB/c mice were divided into 9 PMI time points (0, 2, 6, 8, and 10 days) with 3 mice per time point, and they were exposed to 4 °C and 21 °C. Next, the expression pattern of mmu-miR-133a in the skeletal muscle tissue over a 10-day PMI period was analyzed using the developed molecular beacon probe. **Results:** The molecular beacon (MB) probe was designed for optimal thermodynamic stability with a hairpin structure that opened in the presence of mmu-miR-133a-5p, thus separating the fluorophore from the quencher and resulting in a strong fluorescence signal at 495 nm. Fluorescence intensity increased with mmu-miR-133a-5p concentration from 1 ng/μL to 1000 ng/μL and exhibited a strong correlation (R^2^ = 0.9966) and a detection limit of 1 ng/μL. Subsequently, the expression level of mmu-miR-133a-5p was observed to be stable in mouse skeletal muscle tissue at both 4 °C and 21 °C. **Conclusions:** This user-friendly assay can complete measurements in just 30 min after RNA extraction and is suitable for point-of-care testing, and it possesses the potential to improve existing complex and time-consuming methods for PMI estimation.

## 1. Introduction

In criminal investigations or homicide cases, initial assessment and estimation of the postmortem interval (PMI) are crucial when a body is discovered at a crime scene [1,2]. Accurately estimating the PMI is crucial for profiling suspects and identifying key evidence for solving cases, as it reflects the time elapsed between death and discovery of the body [3]. Over time, different biochemical reactions lead to body decomposition, which can offer valuable information for estimating PMI [4,5].

MicroRNAs (miRNAs) are stable even under extreme conditions and are essential in PMI estimation [6]. miRNAs were discovered by Lee et al. in 1993 as small non-coding RNAs that interact with the 3′ untranslated region (UTR) of target mRNA molecules to regulate post-transcriptional gene expression [7,8,9,10]. miRNAs offer advantages such as size, tissue specificity, resistance to degradation, and excellent sensitivity [11,12,13]. Compared to mRNA, miRNAs are less susceptible to degradation and more stable under various temperature and environmental conditions, thus making them suitable for forensic purposes [6,14,15,16,17]. miRNAs are resistant to postmortem degradation and remain stable even under extreme conditions of temperature, pH, and exposure to various chemicals [18,19,20]. Several studies using miRNAs in mouse or rat models to estimate PMI have reported that changes in the expression of target miRNAs vary with time after death [3,5,21].

Among the various miRNAs, miR-133a-5p was selected in this study due to its well-documented tissue-specific expression in skeletal muscle and its stability, making it a promising candidate for PMI estimation in forensic contexts. Its role in muscle-specific physiological pathways further supports its relevance as a biomarker [21,22]. Furthermore, it has been observed that miR-133 levels decline in liver tissue following death [5], while levels of miR-133a remain consistent in rat myocardial tissue [23].

Molecular beacon (MB) probes were first introduced by Tyagi and Kramer in 1996 and have been widely used in various fields, such as molecular genetics and diagnostics [24,25]. Molecular beacons have been used for sensitive RNA detection in vitro and in vivo [26]. Molecular beacons are oligonucleotide probes (DNA or RNA) with a stem-loop hairpin structure that includes an antisense hybridization sequence flanked by two short self-complementary sequences (typically 4–7 nucleotides). The ends of the probe were linked to a fluorescent dye and an appropriate quencher for that dye [27,28]. In the absence of a complementary miRNA target, the probe maintains a stem-loop structure that causes the fluorescence to be quenched [29]. When the beacon hybridizes with an energetically favorable complementary miRNA sequence, it opens the hairpin probe. This separation of the fluorophore from the quencher allows the fluorophore to emit fluorescence upon excitation. In principle, the probe generates a signal solely upon direct hybridization with the complementary RNA sequence [30,31,32]. As a result, RNA analysis can be conducted in a single step without reverse transcription (RT) or amplification, and excess probes do not need to be removed before measurement. Additionally, they are applicable at a single temperature, thus making them suitable for point-of-care testing (POCT).

**Figure 1:**
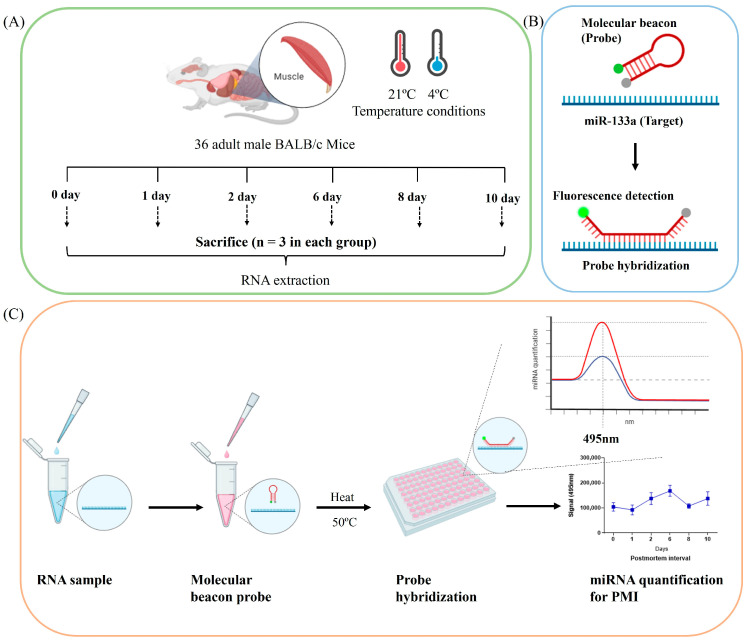
Schematic illustration for detection of mmu-miR-133a-5p using MB to assess PMI. (**A**) Thirty-six healthy adult male BALB/c mice were randomly divided into six groups based on different PMI intervals (0, 1, 2, 6, 8, and 10 days), with each group containing three mice. The mice were exposed to two temperature conditions (4 °C and 21 °C). Skeletal muscle tissues were collected from each mouse, homogenized with TRIzol reagent, and total RNA was extracted. (**B**) The MB probe was engineered for optimal thermodynamic stability in its hairpin structure, but it unfolds in the presence of mmu-miR-133a. (**C**) Steps of the MB probe assay for quantifying mmu-miR-133a. Created in Biorender.

This study used an MB probe assay specific for mmu-miR-133a-5p to detect PMI in skeletal muscle tissues. Additionally, various time intervals were used to investigate changes beyond the previously established 8-day estimate in PMI studies using mouse models. A maximum period of 10 days was used to accommodate the rapid decay process in small mice. The early PMI time points were categorized as 0, 1, and 2 days, while the mid-PMI time points were designated as 6, 8, and 10 days according to previous reports [11,33,34,35] (Figure 1).

## 2. Results

### 2.1. Molecular Beacon Probe Assay for mmu-miR-133a-5p

**Figure 2:**
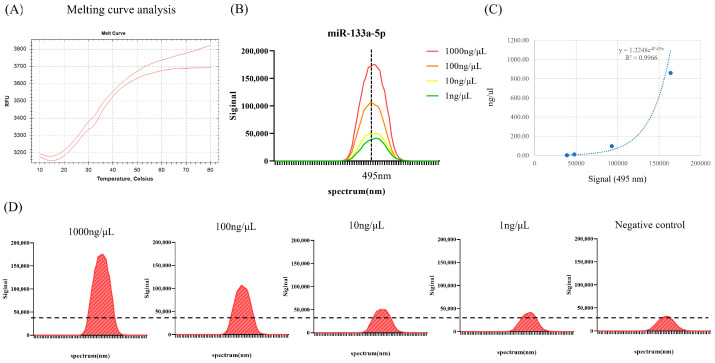
Analytical performance of molecular beacon probe assay for mmu-miR-133a-5p. (**A**) Melt curve of the molecular beacon probe for mmu-miR-133a-5p. (**B**) The fluorescence spectra of the molecular beacon probe in response to varying concentrations of synthetic mmu-miR-133a. (**C**) The standard curve between fluorescence intensity versus the target miRNA concentration. (**D**) Detection limits of the molecular beacon probe assay for mmu-miR-133a-5p using 10-fold serial dilutions of synthetic miR-133a-5p (from 1 ng/μL to 1000 ng/μL). The dotted line represents the limit of detection.

The MB was engineered to achieve optimal thermodynamic stability in its hairpin structure while unfolding in the presence of mmu-miR-133a-5p (Figure 2A, Table 1). The MB was altered to include a fluorophore at the 5′ end and a quencher at the 3′ end. Without mmu-miR-133a-5p, fluorescein (FAM) and the quencher (BHQ1) remained in close proximity. In contrast, when mmu-miR-133a-5p is present, it causes the MB structure to open, thus separating the fluorophore from the quencher and leading to a strong fluorescence signal at 495 nm. As illustrated in Figure 2B, the fluorescence intensity of MB was monitored at various concentrations of mmu-miR-133a-5p. When mmu-miR-133a-5p was absent, the fluorescence of MB was suppressed. However, after adding 1 ng/μL of mmu-miR-133a-5p, an increase in fluorescence intensity was observed. As the concentration of mmu-miR-133a-5p increased from 1 ng/μL to 1000 ng/μL, the fluorescence intensity continued to rise gradually (Figure 2C,D). The relationship between MB fluorescence intensity and different concentrations of mmu-miR-133a-5p is presented in Figure 2C. An excellent correlation (R^2^ = 0.9966) was observed over the range of 0 to 1000 ng/μL, with a detection limit of 1 ng/μL.

### 2.2. Specificity of the Molecular Beacon Probe for mmu-miR-133a-5p

**Figure 3:**
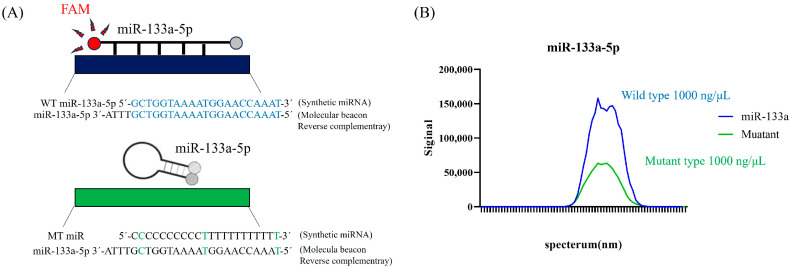
Specificity of the molecular beacon probe assay for mmu-miR-133a-5p. (**A**) A graphical comparison of wild-type and mutant miR-133a-5p used to assess the specificity of the molecular beacon probe assay for mmu-miR-133a-5p. (**B**) The fluorescence spectra of the molecular beacon probe in reactions with wild-type and mutant miR-133a-5p.

To assess the specificity of MB for mmu-miR-133a-5p, the RNA sequence of mature mmu-miR-133a-5p was synthesized. As presented in Figure 3A, a mature miR-133a-5p mutant was used for the hybridization assay. When both wild-type miR-133a-5p and mutant miR-133a-5p were detected using MB, a significant increase in fluorescence was observed with the wild-type miR-133a-5p (Figure 3B).

### 2.3. Detection of mmu-miR-133a-5p Using a FAM-Labeled MB Probe for PMI

**Figure 4:**
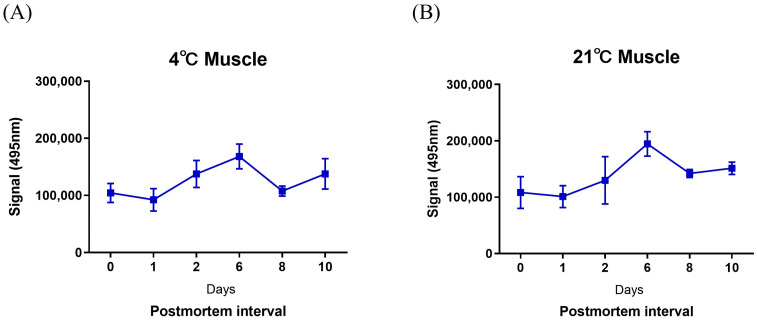
Detection of mmu-miR-133a-5p using the MB probe at different time intervals at (**A**) 4 °C or (**B**) 21 °C in mouse skeletal muscle. Data are presented as mean ± standard error of the mean.

The levels of mmu-miR-133a-5p were assessed using the MB in the skeletal muscle tissues of 36 adult male BALB/c mice to evaluate their effectiveness for identifying prolonged postmortem intervals (PMIs) ranging from 0 to 10 days (0, 1, 2, 6, 8, and 10 days; Figure 4). The expression of mmu-miR-133a remained consistent in skeletal muscle tissue at both low (4 °C) and high (21 °C) temperatures.

## 3. Discussion

The precise estimation of the PMI is crucial for profiling suspects and uncovering key clues to solve a given case, as the PMI indicates the time between death and discovery of the body [36]. Various methods have been employed to estimate PMI, including visible signs such as decreases in body temperature, rigor mortis, and livor mortis. Additionally, biochemical techniques such as decay indices, blood color changes, rectal temperature measurements, thermal imaging, and forensic entomology are used to accurately estimate PMI [37,38]. Over time, different biochemical reactions cause the body to decompose, providing data for estimating the PMI. Research focused on mRNA, DNA, and protein analyses for PMI estimation is ongoing [39]. Among these, miRNA is regarded as a critical factor for PMI estimation due to its stability under extreme conditions [3,5,18,19,20,23]. Therefore, we assessed the potential of miRNAs as molecular markers for PMI estimation in a mouse model subjected to various temperatures and times.

In the search for better methods to detect and quantify miRNAs, molecular beacons have emerged as promising tools due to their advantageous properties for nucleic acid detection. Their operational principle relies on the stem-loop structure that brings the fluorophores at their ends close together due to the complementarity of the stem sequences [29]. When the target binds to the MB due to this complementarity, the interaction causes the stem region to separate. This separation of the stem sequences induced by the hybridization of the target with MB moves the fluorophores apart, thus producing a fluorescence signal [40]. In this study, a fluorescent MB probe with high specificity for mmu-miR-133a-5p was developed based on its distinct sequence. Under optimized conditions, the hybridization assays demonstrated promising linear range, sensitivity, and practical applicability for targeting miRNAs, particularly in biological samples. The newly designed MB probe assay was assessed based on analytical performance parameters including melting, standard, and sensitivity and specificity. Melting curve analysis confirmed that as the temperature increased, the MB structure unfolded, thus causing the fluorescent dye at one end to move away from the quencher and emitting a fluorescence signal. Furthermore, a strong correlation (R^2^ = 0.9966) was observed between the fluorescence intensity of the MB and different concentrations of mmu-miR-133a-5p within the range of 0 to 1000 ng/μL, with a detection limit of 1 ng/μL. Next, we investigated the expression patterns of mmu-miR-133a-5p in skeletal muscle tissue over 10 days utilizing a newly developed MB probe and spanning different PMI time points. mmu-miR-133a maintained its stability in skeletal muscle tissue for as long as 10 days at both 4 °C and 21 °C. Similarly, Tu et al. confirmed that mmu-miR-133a remains stable in skeletal muscles up to day 8 [35]. The stability of miR-133a expression may be due to its role in muscle development and function, making it less vulnerable to environmental fluctuations [41].

Using the MB probe assay can be performed in a single step, bypassing the need for reverse transcription (RT) or amplification, and it does not require the removal of excess probes before measurement. Additionally, it is advantageous for point-of-care testing (POCT) due to its single-temperature applicability [42,43]. The MB probe assay developed in this study is also notably user-friendly and completes measurements within 30 min of RNA extraction. Alternative isothermal methods, such as Loop-Mediated Isothermal Amplification (LAMP), have shown promise for miRNA detection due to their sensitivity and ability to operate at constant temperatures [44]. However, LAMP protocols typically require reverse transcription and complex primer sets, which can increase preparation time and contamination risk [45]. In contrast, the molecular beacon (MB) probe assay developed in this study directly hybridizes with mmu-miR-133a-5p without the need for reverse transcription, providing a streamlined, single-step process that minimizes contamination risks. The high sequence specificity of MB probes also reduces the likelihood of off-target hybridization, a common issue with LAMP. Although LAMP-based miRNA detection has seen limited application in postmortem interval (PMI) estimation within forensic science, future studies could compare MB and LAMP systems to further emphasize the advantages of MB technology for rapid and precise forensic applications. While we chose the MB probe system for its simplicity and speed, eliminating the reverse transcription and amplification steps required by quantitative polymerase chain reaction (qPCR), we acknowledge that qPCR remains a highly sensitive and robust method for miRNA detection [6,13,15]. Comparing our MB assay with qPCR will provide valuable insights into the sensitivity of these techniques, and future investigations will aim to evaluate the efficacy of the MB probe system and its potential utility in forensic contexts.

In this study, mmu-miR-133a-5p stability was evaluated under controlled temperature conditions (4 °C and 21 °C) to simulate environments relevant for forensic analysis. However, it is important to note that miRNA stability may vary in more complex settings, such as contaminated or microbially active environments. In such cases, the presence of microbial enzymes could accelerate RNA degradation, potentially affecting miRNA stability and, consequently, the robustness of forensic assays. Notably, existing studies indicate that miRNAs, including miR-133a-5p, tend to exhibit greater resistance to environmental degradation than other RNA types due to their small size and secondary structure, making them suitable markers for forensic timelines [14,15,16,17,18,19,20]. Nevertheless, further research on miRNA stability in contaminated environments would be beneficial to delineate the full range of conditions under which miR-133a-5p can reliably function as a forensic marker. Additionally, we recognize that miRNA profiles vary across tissues and can be altered by pathological conditions, like hepatic cancer, which specifically affects miR-133 expression in the liver [46,47]. These differences emphasize the importance of tissue-specific miRNA markers for forensic accuracy.

The observed variations in miRNA expression patterns in this study underscore the complexity of molecular changes occurring postmortem and the challenges associated with using miRNAs for PMI estimation. The limited sample size in our study constrains the statistical power and generalizability of the findings, necessitating further validation through larger cohorts. Moreover, while this study focused on controlled temperature conditions (4 °C and 21 °C), real-world forensic environments often involve a broader range of variables, such as humidity, body mass, and exposure to natural elements, which could influence miRNA stability. These factors should be examined in future research to enhance the robustness and applicability of miRNA-based PMI estimation methods.

Importantly, our findings were derived from animal models, which may not directly apply to human forensic scenarios. Notably, the molecular beacon (MB) probe used in this study was specifically tailored for mouse miR-133a-5p (mmu-miR-133a-5p). The sequence of mmu-miR-133a-5p (GCUGGUAAAAUGGAACCAAAU) differs by a single nucleotide from the human version, hsa-miR-133a-5p (AGCUGGUAAAAUGGAACCAAAU), as sourced from miRBase. This subtle difference may influence cross-species applications, underscoring the necessity of species-specific probe design. These considerations highlight the importance of further studies involving human samples to validate miR-133a-5p as a postmortem interval (PMI) marker while accounting for biological and species-specific factors, such as genetic variability and age.

In conclusion, MB probe analysis possesses the potential to enhance existing complex and time-consuming methods, such as those involving target amplification for PMI estimation, and it is suitable for point-of-care testing.

## 4. Materials and Methods

### 4.1. Sample Collection

A total of 36 healthy adult male BALB/c mice were procured from KOSA BIO Inc. (Seongnam, Republic of Korea) and randomly allocated to 9 different postmortem interval (PMI) time points, including 0, 2, 6, 8, and 10 days, with 3 mice per time point.

The mice were exposed to two different temperature conditions (4 °C and 21 °C). For each group, skeletal muscle tissue was collected from each mouse, weighed, and then stored at −80 °C. This study was approved by the Animal Ethics Committee (AEC) of the Catholic University of Pusan (approval number: CUP AEC-2022-001).

### 4.2. Extraction of Total RNA

All tissue samples were homogenized using TRIzol reagent (Invitrogen, Waltham, MA, USA), and total RNA was extracted according to the manufacturer’s guidelines.

The collected tissues were separated into microtubes with 50 and 100 mg aliquots. TRIzol (1 mL) was added to each microtube containing the tissue samples, and the mixture was homogenized using a homogenizer. The homogenized sample was vortexed for 15 s, and this process was repeated 10 times. Samples were incubated for 5 min to facilitate the breakdown of nuclear proteins. After incubation, chloroform (0.2 mL per 1 mL of TRIzol) was added, and the mixture was shaken vigorously for 15 s. Following a 3 min incubation at room temperature, the samples were centrifuged at 12,000× *g* for 15 min at 4 °C. After centrifugation, the samples were separated into three layers that included the organic phase (containing proteins and lipids), the interphase (containing DNA), and the aqueous phase (containing RNA). Only the uppermost aqueous phase was carefully transferred to new 1.5 mL tubes. An equal volume of isopropanol was added to the aqueous phase, gently inverted six times, and then incubated at −80 °C for 30 min. After incubation, the samples were centrifuged at 12,000× *g* for 10 min at 4 °C. The supernatant was discarded, and the RNA pellet was retained. RNase-free water (20–50 μL, DEPC-treated) was carefully added, and the mixture was incubated at 55 to 60 °C on a heat block for 10 min. Finally, the concentration and purity of the extracted total RNA were assessed using a NanoDrop 2000 spectrophotometer (Thermo Fisher Scientific, Waltham, MA, USA), and the samples were stored at −80 °C for future use.

### 4.3. Design of a Molecular Beacon for mmu-miR-133a-5p

A molecular probe was developed to complement the mature nucleotide sequence of mmu-miR-133a-5p. These beacons included a minimum of 25 complementary bases, with a 6-FAM fluorophore attached to the 5′ end and a Black Hole Quencher 1 (BHQ1) at the 3′ end. Molecular beacons were synthesized by Integrated DNA Technologies (IDT; Coralville, MD, USA) and purified using High Performance Liquid Chromatography. They were dissolved in nuclease-free water to a concentration of 1 mg/mL, transferred to an opaque tube, and stored at −20 °C.

To assess the sensitivity and specificity of the molecular beacons, the RNA sequences of miR-133a-5p wild type (WT) and miR-133a-5p mutant type (MT) were synthesized by IDT (Table 1).

### 4.4. Beacon Hybridization Assay

Hybridization between the MB and mmu-miR-133a-5p was assessed in vitro at 50 °C, with fluorescence intensity measured using a VarioskanTM LUX Multimode Microplate Reader (Thermo Fisher Scientific, Waltham, MA, USA). Molecular beacons and synthetic miRNA were combined in DEPC, resulting in a final volume of 100 µL and a beacon concentration of 1 pmol. The mixtures were incubated at 50 °C for 30 min in a 96-well flat-bottom tray before measuring the fluorescence intensity. The fluorescence intensity of the MB probe was measured between 450 nm and 520 nm using the Varioskan™ LUX.

### 4.5. Statistical Analysis

Statistical analyses were performed using GraphPad Prism (version 8.0) and SPSS Statistics (version 21.0; IBM, Armonk, NY, USA). A *p*-value of less than 0.05 was considered statistically significant for all analyses.

## Figures and Tables

**Table 1:** Sequence and molecular beacon probe specific for mmu-miR-133a-5p that was used in this study.

Probe	Sequence	Dye	Quencher	Probe Length	Stem Length	Tm (°C)
MB for mmu-miR-133a-5p	5′-ATTTGGTTCCATTTTACCAGCAAAT-3′	FAM	BHQ1	25	5	54
mmu-miR-133a-5p (target)	5′-GCTGGTAAAATGGAACCAAAT-3′					
Mutant miR-133a-5p	5′-CCCCCCCCCCTTTTTTTTTTTT-3′					

MB, molecular beacon.

## Data Availability

The data generated or analyzed during this study are included in this article. Some of the datasets are available from the corresponding author upon reasonable request.
